# Synergy of Nitric Oxide and 1-Methylcyclopropene Treatment in Prolong Ripening and Senescence of Peach Fruit

**DOI:** 10.3390/foods10122956

**Published:** 2021-12-01

**Authors:** Xiaoqin Wu, Jiawei Yuan, Xiaoqing Wang, Mingliang Yu, Ruijuan Ma, Zhifang Yu

**Affiliations:** 1 College of Biological and Food Engineering, Changshu Institute of Technology, Suzhou 215500, China; wuxiaoqin@cslg.edu.cn (X.W.); yuanjiawei5008@163.com (J.Y.); w1522079778@163.com (X.W.); 2 College of Food Science and Engineering, Nanjing Agricultural University, Nanjing 210095, China; 3 Institute of Pomology, Jiangsu Academy of Agricultural Sciences/Jiangsu Key Laboratory for Horticulture Crop Genetic Improvement, Nanjing 210014, China; marj311@163.com

**Keywords:** fruit storage, antioxidant capacity, phenolic compounds, gene expression

## Abstract

Peach is a putrescible fruit thus drastically restricting its postharvest storage life. In recent years, the application of 1-methylcyclopropene (1-MCP) and nitric oxide (NO) in postharvest fruit quality control has received considerable attention and investigative efforts due to the advantages of using relatively low concentrations and short-time treatment duration. In the present study, the effects of various 1-MCP and NO treatments on peach fruit (*Prunus persica* L. cv. Xiahui-8) stored at 25 °C were evaluated and compared. Results indicated that the combination treatment with both chemical agents (MN) was most effective in postponing peach ripening and preserving fruit quality, followed by 1-MCP and NO treatment alone. We also demonstrated that NO could delay fruit senescence mainly by stimulating antioxidant enzymes, while 1-MCP overly outperformed NO in the treatment of ‘Xiahui-8′ peach in slowing down respiration rate, inhibiting ethylene production, maintaining high firmness and reducing ROS content. NO treatment showed a greater influence on phenolic compounds than 1-MCP especially anthocyanins, flavanones and flavones according to LC/MS analysis. The phenolic change in MN group were highly associated to NO treatment. Through this study we provide informative physiological, biochemical and molecular evidence for the beneficial effects of the combined 1-MCP and NO treatment on peach fruit based on a functional synergy between these two chemical agents.

## 1. Introduction

Unlike other rosaceous fruit like apple or pear, peach (*Prunus persica* L.) is well known for the relatively short shelf-life of fruits due to high respiration rates, accelerated fruit ripening and fast flesh softening process that could significantly impede marketing and sales and lower commercial value. Hence, there is an urgent need to develop effective strategies for postharvest handling and storage in order to prolong shelf-life while maintaining consumer-desired fruit quality. Towards this goal, several previous studies investigated the use of 1-methylcyclopropene (1-MCP) and nitric oxide (NO) for postharvest treatment in peach fruits and demonstrated their high efficacy for delaying fruit ripening and senescence at relatively low concentrations and short treatment time duration [1,2,3].

The biological function of NO as a key signaling molecule in plant cells has long been recognized. For instance, Neill et al. in their investigation of the molecular events related to NO biosynthesis and functionality demonstrated that NO produced by plant cells can function as a critical signaling component in ABA-induced stomatal closure [4]. Fumigation treatments of climacteric and non-climacteric fruits with NO, later known as an ethylene antagonist, were found highly effective to considerably extend fruit postharvest life and delay senescence [5,6]. Likewise, another ethylene inhibitor 1-MCP has also been utilized in the postharvest treatment of fruits and vegetables due to its pronounced effects to dramatically delay ripening, lower ethylene production and respiratory rate and maintain desirable quality [1]. With these attractive properties, the utilization of either 1-MCP or NO in postharvest treatment of peach has been widely attempted [1,2,5,7,8]. However, to the best of our knowledge, no reports are available that describe the combined use of these two chemical agents and investigate if there are any synergistic effects as compared to single chemical treatment on peach ripening and senescence.

It is worth noting that the modes of action of these two chemical agents are strikingly different. 1-MCP is a competitive inhibitor of ethylene perception and is capable of interacting with ethylene receptor sites and thus preventing the ethylene-induced signaling that triggers ripening and senescence [9]. On the other hand, NO constitutes an important component in the endogenous signaling pathway in cellular metabolism and functions to modulate the physiological responses to phytohormones [10]. The fate of peach fruits upon treatment with both chemical agents remain unknown. Therefore, the objective of this work was to evaluate the effects of peach postharvest treatment with 1-MCP and NO individually or in combination and reveal the patterns of physiological response and gene expression associated with the treatments in order to explore better options for controlling ripening and decline in postharvest fruit quality.

## 2. Material and Methods

### 2.1. Peach Material and Treatment

Peach fruits (*Prunus persica* L. cv. Xiahui 8) were harvested from an orchard at Jiangsu Academy of Agricultural Sciences (JAAS) in Nanjing, Jiangsu, China. After 120 days post florescence, about 600 peaches with uniform size and without obvious defects or damages were picked and placed in a pre-cooled container, then transported to the lab immediately. The collected fruit were randomly divided into four groups and subjected to the following treatments: (1) CK or control group: 150 fruit were directly stored at 25 ± 2 °C with 85–90% humidity for 8 days; (2) N group with NO treatment: 150 fruit were placed in a sealed container and treated with 10 μL L^−1^ NO gas for 3 h [2]; (3) M group with 1-MCP treatment: 150 fruit were transferred to an enclosed container and treated with 10 μL L^−1^ 1-MCP (Sinopharm Chemical Reagent Beijing Co., Ltd., Beijing, China) for 12 h, and 1% (*w/v*) KOH solution was placed inside to prevent CO_2_ accumulation [1]; (4) MN group with the combination of 1-MCP and NO treatments as described in published literature [11]: 150 peach fruit were first treated using the same condition as 1-MCP treatment for 12 h, and then subject to fumigation with 10 μL L^−1^ NO gas for 3 h. All fruits were stored at room temperature (25 ± 2 °C) with 80–90% relative humidity for 8 days. Samples were taken at 0, 2, 4, 6, and 8 days during storage and immediately used for measurement of respiration, ethylene production, firmness, total soluble solid (TSS), titratable acid (TA), H_2_O_2_, malondialdehyde (MDA) and O_2_^−^ content. The rest of the fruits were peeled to remove skin and cut into pieces, frozen with liquid nitrogen and stored at −80 °C for further analysis. For each time point, 30 fruit samples were employed for each of three biological replicates, and only the mesocarp was used for analysis.

### 2.2. Respiratory Rate and Ethylene Production

For respiration and ethylene production, fifteen fruit were placed in three airtight containers equally for 1 h. CO_2_ production rate was measured by a portable infrared CO_2_ analyzer (PBI Dansensor CheckMate 3, Copenhagen, Denmark) and respiration rate was expressed as mg kg^−1^ h^−1^ of CO_2_. Ethylene production was performed according to a method described by Huan [12], with minor modifications. One milliliter of the headspace gas was taken out form each jar and injected into a gas chromatograph (Agilent GC7890 A, Palo Alto, Santa Clara, CA, USA) equipped with an HP-AL/S column (30 m × 0.53 mm × 15 μm, Agilent, Palo Alto, Santa Clara, CA, USA) and a flame ionization detector (FID). The injector, oven and detector temperatures were 120, 100 and 200 °C, respectively. Ethylene production was expressed as µg kg^−1^ h^−1^.

### 2.3. Firmness, MDA, H_2_O_2_ and O_2_^−^ Detection

For fruit firmness, 10 fruit were used and evaluated by using a Fruit Hardness Tester (FHM-5, Tokyo, Japan). MDA, H_2_O_2_ and O_2_^−^ were measured according to our previous report [13] and expressed as mmol per kilogram fresh weight (mmol kg^−1^ FW).

### 2.4. Enzymatic Assays

Activities of total superoxide dismutase (SOD) and catalase (CAT) were assayed as described in our previous report [14]. Peroxidase (POD) activity was measured according to the method of Zhang et al. [15] with minor modifications. Following steps were used for the assay: the reaction mixture was prepared by combining guaiacol (0.25%, 100 μL), crude enzyme extract (50 μL) and acetic acid buffer (100 mM, pH 5.4, 100 μL); the reaction was initiated by adding 50 μL of H_2_O_2_ (0.15%); and absorbance of the sample at 460 nm was measured. Polyphenol oxidase (PPO) activity was determined according to Yingsanga et al. [16]. Ascorbate peroxidase (APX) activity was assayed according to the method of Song et al. [17]. The absorbance changes of POD, PPO and APX reaction mixtures were measured using Microplate Reader (Tecan, Switzerland) for an assay duration of 6 min. One unit of these enzyme activities was defined as a change of 0.01 in absorbance per min and activities expressed as U per mg protein. PAL activity was assayed according to preciously published protocol [18]. Protein content in the extracts was determined by reading absorbance of the sample at 595 nm via the method of Bradford [19] using bovine serum albumin (BSA) as a standard.

### 2.5. RNA Isolation and Gene Expression Analysis

Sequence information on genes encoding POD and PAL was derived from Genome Database for Rosaceous (GDR; http://www.rosaceae.org/peach/genome (accessed on 16 October 2021)) and gene specific primers were designed using Primer 5.0 software and used for transcript sequencing. After screening of received sequencing data and discarding the redundant sequences, two *PpaPODs* and one *PpaPAL* were selected for further analysis. Primers for *PpaSOD*, *PpaCAT* and *PpaAPX* that were designed in previous research using the similar cultivar [20] were utilized herein. A translation elongation factor 2 (*PpaTEF 2*) was selected as s reference gene for its high expression stability [21]. All primers used for this study were showed in Supplementary Materials. Total RNA extraction, first-strand cDNA synthesis and real-time quantitative reverse transcription-polymerase chain reaction (qRT-PCR) analysis were performed according to our previous report [22].

### 2.6. LC/MS Analysis of Phenols

The obtained data manifested that respiration burst on D4 and D8, meanwhile ethylene production reached the peak on D8. We speculate that D4 and D8 is crucial time of peach fruit metabolism. Therefore, we choose peach materials of these two time point for further LC/MS analysis. The phenolic compounds extraction and LC/MS analysis were conducted followed by our previous report [22]. Briefly, approximately 10 g of peach tissue was ground with liquid nitrogen, then accurately weigh 5 g of ground sample and homogenized in 100 mL of 95% acidic (0.1 M HCl) methanol. After 4 h of extraction, the mixture was centrifuged at 10,000× *g* for 20 min. The supernatant was collected and evaporated to dryness. For LC/MS analysis, the residue was redissolved in 6 mL of methanol and filtered through a 0.22 μL membrane (Millipore) filter. LC/MS analysis system (G2-XS QTof, Waters) and liquid chromatography (UPLC) column (2.1 × 100 mm × 1.7 μm) was used in this study according to our previous research [22].

### 2.7. Statistical Analysis

The experiment was conducted in a completely randomized design. Figures were made with Origin Pro 2017 software program. Statistical analyses were performed with the SPSS 18.0 software using the Duncan’s test with a significance level at *p* < 0.05. Pearson correlation test were performed with SPSS software.

## 3. Results and Discussion

Based on extensive characterization of the role in reducing aging of cut carnations, the use of 1-MCP as an ethylene antagonist in postponing the ripening of edible fruits and vegetables has been proposed previously [23]. Subsequently, numerous studies have shown that 1-MCP can extend postharvest life of a wide variety of food commodities [9]. NO was revealed to act as an important endogenous signaling molecule in many cellular metabolisms to modulate hormonal homeostasis during stress responses and plant developmental processes [10]. However, the detailed mechanisms of action through which NO affects fruit ripening and storage quality remain unclear. Other research reports have shown that free radical gas NO has anti-senescence properties similar to those of 1-MCP, which has been observed in tests with different fruits and vegetables [7]. This presumptive finding was somewhat confirmed in our investigations according to results of the following indices.

### 3.1. Firmness

**Figure 1:**
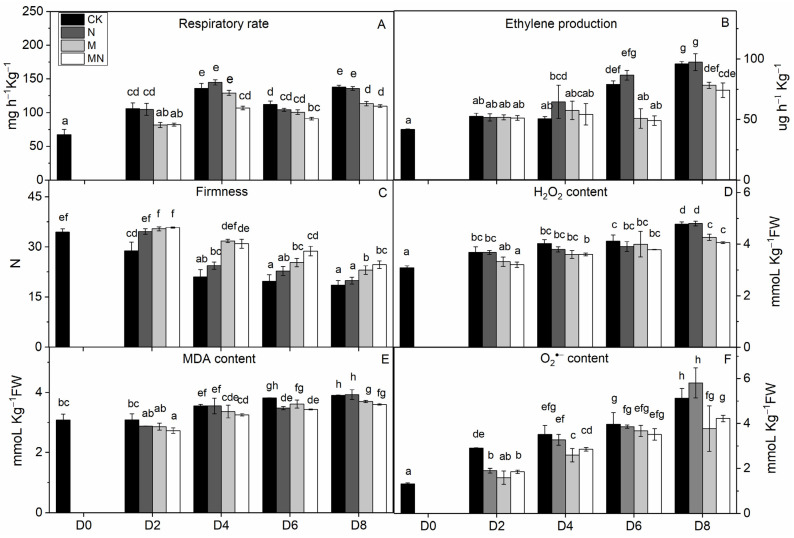
Physiological and biochemical indices of peach fruit including respiratory rate (**A**), ethylene production (**B**), firmness (**C**), H_2_O_2_ content (**D**), MDA content (**E**) and O_2_^−^ content (**F**) used in this research. Each point represents means ± SE of three replicates. The lowercase letters indicate significant differences according to statistical analysis.

Firmness is an important quality attribute of peaches that has the potential to enhance storage potential, improve resistance to decay organisms and mechanical injury and enhance market appeal and consumer preference [24]. Fruit softening is a consequence of the modifications and content changes of different cell wall polymers, which is a natural physiological process during ripening and senescence. In this work, firmness was excellently maintained by MN treatment followed by the 1-MCP treatment. Firmness value in NO treatment had no significant change since D4 to the end of storage as compared to that in CK (Figure 1C). Results showed that 1-MCP treatment alone or combined treatment can maintain high firmness of peach, which can delay fruit ripening by maintaining cell structure and improving resistance of decay organisms. NO application can inhibit flesh softening process at later storage time, but which effect was not superior to another two groups in this study.

### 3.2. Respiration and Ethylene Production

Fruit respiration converts storage compounds and sugars to energy via the generation of ATP to maintain normal metabolism. Respiration and ethylene production are critical indicators of peach ripening. As climacteric fruit, peach is characterized by an upsurge in the respiration rate coincided with a burst of ethylene production during ripening stage. In this research, the respiration rate showed a normal feature of climacteric fruit, which reached a respiratory peak at D4, thereafter decreased (Figure 1A). However, the respiration at D8 showed a high value, which might be induced by tissue damage in later stages of fruit senescence [25]. The ethylene release increased throughout the storage time, reaching a maximum level at D8, a trend consistent with previous studies [20]. 1-MCP or MN treatment significantly suppressed the ethylene release from D6 to the end of storage, but NO treatment had no distinctive effect on ethylene production throughout the storage duration (Figure 1B). Besides, the onset of ethylene productive peak was falling significantly behind respiratory climacteric peak, a phenomenon similar to what was reported in other research with peach [26]. MN treatment suppressed both respiratory and ethylene production rates to the lowest level, indicative of a better approach to postponing fruit ripening. Noticeably, 1-MCP treatment had a better effect in slowing down respiratory and ethylene production rates than NO treatment. We postulate that 1-MCP may be more efficient in delaying senescence of this cultivar. The results of combined 1-MCP and NO treatment observed herein were consistent with previous research with blueberry fruit [11], in which similar combination treatment significantly extended the postharvest life of one of the two compared blueberry cultivars. Several studies have also demonstrated that NO could inhibit CO_2_ and ethylene production [7,27]. In particular, Zhu and coworkers theorized that NO is bound to 1-aminocyclopropane-1-carboxylate (ACC) oxidase and subsequently chelated to ACC to form an ACC-ACC oxidase-NO complex, thus decreasing enzymatic activity and reducing ethylene production [27]. NO at 10 μL/L was shown to exert excellent effect on a peach cultivar ‘Feicheng’ [27]. In spite of these reports, however, we found that treatments with NO at various concentrations on peach cultivar ‘Xiahui-8′ did not yield desirable results based on observation in several physiological indices. Furthermore, no statistical difference of ethylene release was observed between N and CK groups. These findings may reflect the distinct genotypic response of different cultivars to NO treatment with mechanisms thus far remained unknown. 

### 3.3. Reactive Oxygen Species (ROS) Production

Peach is putrescible fruit and can soften quickly at normal temperature, which makes it particularly vulnerable to internal and external stresses. When there is a serious imbalance in cell compartment between the production of ROS and antioxidant defense or ROS scavenging during peach ripening, the ROS increase will inevitably occur, leading to oxidative damage to many biological macromolecules, including proteins, DNA and lipids [14]. ROS can cause peroxidation of the membrane lipids resulting in cell membrane alterations and consequently the generation of MDA [7]. In this study, the tendencies of H_2_O_2_, MDA and O_2_^−^ production were all similarly increasing gradually throughout the entire duration of storage (Figure 1), indicating that oxidative stress takes place during the natural course of ripening and senescence. The effect of 1-MCP or NO application alone on ROS reduction were mentioned in various researches such as apple [28], mango [29], winter jujube [30]. However, the comparison between these two treatments on peach fruit haven’t not been reported yet. Our results showed that, as compared with control fruit, combination treatment MN or treatment with 1-MCP alone significantly reduced the production of ROS, thus delaying fruit senescence. For explaining the mechanism underlying this phenomenon, Lin [31] presumed that postharvest treatment could alleviate the damage action of ROS and the peroxidation process of membrane lipids, consequently retain the structure of pulp cellular membrane of fruit. Combined with the results we got, we assumed that the raised level of ROS and MDA content were highly related to the breakdown of cell structure (Figure 1C–F).

### 3.4. Enzymatic Activity

**Figure 2:**
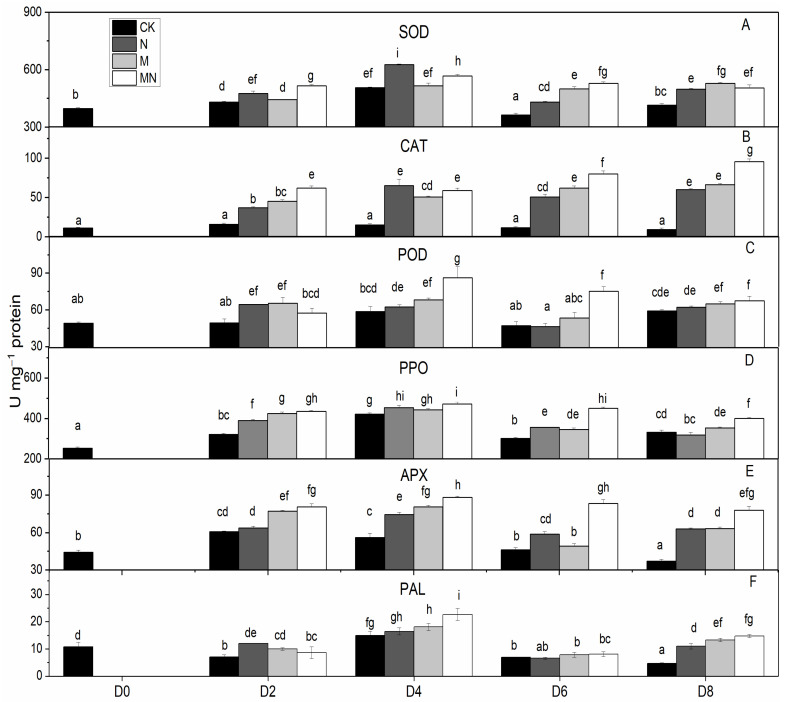
Enzymatic activity in peach fruit. SOD activity (**A**), CAT activity (**B**), POD activity (**C**), PPO activity (**D**), APX activity (**E**), PAL activity (**F**). Each point represents means ± SE of three replicates. The lowercase letters indicate significant differences according to statistical analysis.

Effective reduction of ROS requires several antioxidant enzymes such as SOD, CAT, POD and APX. These enzymes act concomitantly with non-enzymatic antioxidants as a defense against excess ROS [32], consequently, inhibiting fruit quality deterioration. SOD is the first line of defense against ROS by catalyzing the dismutation of O_2_^−^ to molecular oxygen and H_2_O_2_, and H_2_O_2_ is then scavenged by CAT, POD and APX [33]. In fruit, these antioxidant enzymes are well known for their roles in regulating the accumulation of ROS which can also act as signaling molecules in many biological processes, and recent studies showed that they are also involved in regulating fruit development [34] and ripening [35]. Furthermore, these antioxidant enzymes are readily activated by postharvest treatments such as hot water [20], brassinolide [36] and 1-MCP [37], which can effectively scavenge ROS to extend the shelf-life as well as improve fruit chilling tolerance [20]. In this study, similar trends of total SOD, APX, PPO and POD activities were observed for all treatments in contrast to CK (Figure 2): a discernable increase for the first four days (D0 to D4), followed by a slight decline (D6), and then a small increase at the end of observation period (D8). CAT activity in treated fruit was noticeably activated than untreated fruit (CK) with a further increased level at D8 during ripening (Figure 2B). Overall, MN treatment induced the highest enzymatic activities for all examined enzymes than those of the control during the entire storage with the exception of total SOD and CAT activities at D4, in which NO treatment generated higher levels. 1-MCP showed better effects for enhancing enzymatic activity than NO, suggesting the former is more effective in ROS elimination. Previous studies showed that exogenously applied NO increased the activity of total SOD, CAT and APX [38,39]. Our results consistent with pervious findings and showed enhanced enzymatic activities of these enzymes. However, the enzymatic activity of total SOD, CAT and APX as well as POD in N group most of the time were not superior to those in M group. Considering that a higher respiratory rate was observed in N group, the lower levels of activity of antioxidant enzymes could lead to increased accumulation of ROS. It is interesting that the general trends of total SOD, APX and POD activities are the same in all treated fruit, i.e., reaching the maximum level at D4 thereafter followed by a gradual decrease, and is similar with respiratory tendency. On the other hand, CAT activity continually increased till the end and did not show any peaks. Similar results for total SOD and CAT activities were also reported in previous studies with peach [40]. We speculate that high respiratory rate generates more ROS, which will in turn stimulate total SOD, APX and POD activities [20,41].

PAL is the first key enzyme in biosynthetic pathway of phenols in fruit and can be induced under various stress conditions [42]. PPO catalyzes the hydroxylation of monophenols that results in brown pigments. In this study, PPO and PAL activities manifested a similar changing trend in four groups, while maintaining a highest level at D4. MN outperformed individual NO or 1-MCP treatment in enhancing PPO and PAL activities, whereas untreated fruit (CK) possessed the lowest activity (Figure 2). It has previously been reported that exogenous NO can stimulate antioxidant enzymes such as PAL [43,44] and POD [43,45], which is in accordance with the results found in this study. However, unlike our results, 1-MCP application on strawberry [46], loquat [47] and nectarine [48] was found to inhibit activities of PAL and PPO and therefore was employed to prevent fruit browning. We speculate that the increased activities of PAL and PPO observed in our study were attributable to resistance response to biotic and abiotic stress processes [49,50].

### 3.5. Gene Expression Analysis

**Figure 3:**
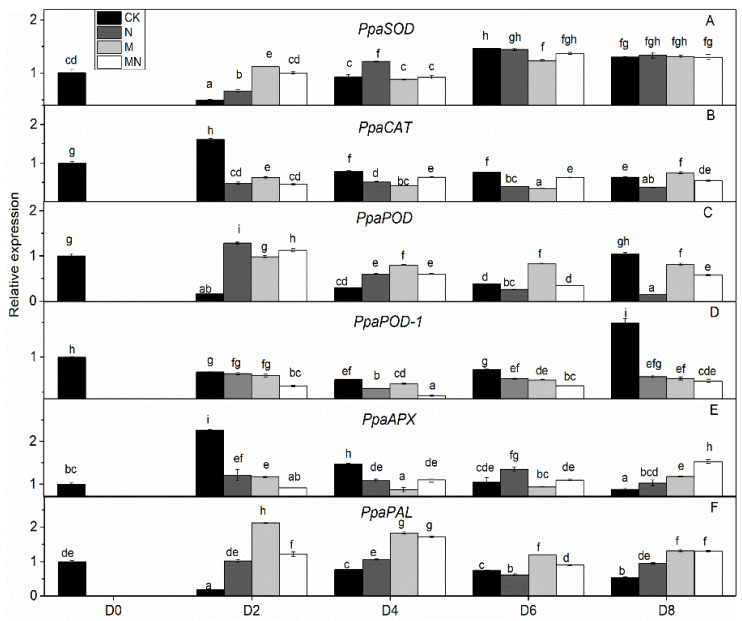
Gene expression profile in peach fruit including *PpaSOD* (**A**), *PpaCAT* (**B**), *PpaPOD* (**C**), *PpaPOD-1* (**D**), *PpaAPX* (**E**) and *PpaPAL* (**F**). Each point represents means ± SE of three replicates. The lowercase letters indicate significant differences according to statistical analysis.

In order to check whether trends of enzymatic activity and related gene expression were similar or not in a quantitative way, we did Pearson correlation test, and the result are showed in Table 1. Through the values we found that almost all the genetic and enzymatic changes were inconsistent with each other except *PpaPAL*/PAL (*p* < 0.01). Similar results can be seen in series of published papers [3,51]. We postulate that these enzymes might be regulated by different, yet unidentified, gene members and factors. As showed in Figure 3, *PpaCAT* and *PpaAPX* exhibited a similar expression pattern across all treatments, while their levels of expression in CK were higher than that of treated fruit and at the same time followed a decreasing trend during storage time. Expression of *PpaSOD* declined at D2 then followed a continuous increase during the period from D4 to the end D8. No distinguishable patterns of change in the expression of these genes were observed amongst all three treatments.

The relative expression levels of *PpaPAL* in treated fruit were higher than that of untreated one (CK), and 1-MCP treatment or combined 1-MCP and NO treatment induced higher expression levels than NO treatment. Additionally, both PAL activity and *PpaPAL* expression exhibited a similar changing trend in response to postharvest treatments and storage process. Accordingly, the Pearson correlation value of *PpaPAL* and PAL was 0.588 (*p < 0.01*), which indicated that treatments tested herein might stimulate PAL activity by directly promoting the expression of *PpaPAL*.

Different trend patterns were observed between two POD genes: *PpaPOD* and *PpaPOD1* (Figure 3). The expression tendency in CK remained the same for these two genes, which decreased at first thereafter increased from D4 till the end D8. 1-MCP treatment stimulated *PpaPOD* expression from D2 to D6, thereafter maintained a stable expression level. The treatment methods showed a greater impact on the expression of *PpaPOD* than *PpaPOD1*, while *PpaPOD1* expression levels in variously treated fruit remained consistent with little changes, but all lower than that in CK group. In addition, similar dynamic changes in the levels of POD and *PpaPOD* expression indicated that POD enzyme might be regulated directly by expression activity of *PpaPOD*.

### 3.6. LC/MS Analysis of Phenols

**Figure 4:**
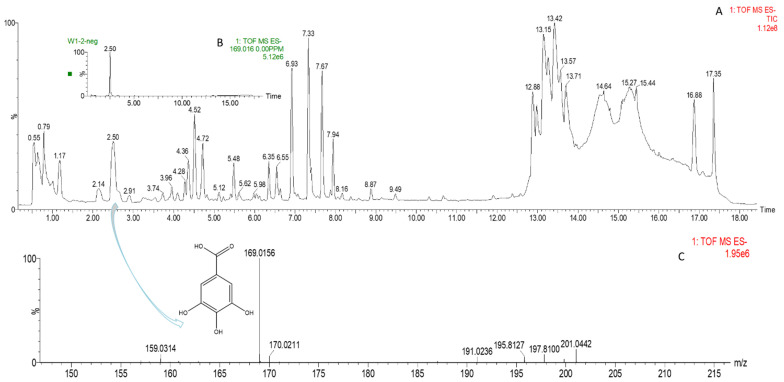
(**A**)Representative mass spectrogram of phenolic compounds from peach tissue extract from m/z 100 to 1000 (negative ionization mode); (**B**) mass spectrogram (MS) of galic acid at m/z 169.016; (**C**) MS/MS at 2.50 RM (negative ionization mode).

20 phenolic compounds were successfully identified based on their retention times, MS data and the corresponding specific fragment, including anthocyanins, flavanones, flavanols, flavones, flavonols and phenolic acids. Representative mass spectrogram of galic acid at negative ionization mode were showed in Figure 4. We have already investigated the influence of 1-MCP on phenolics in our previous report [22], and here we emphasize the effect of NO treatment alone and the combined treatment. The relative amount of phenols were showed in Table 2. Four kinds of anthocyanins were successfully detected including Pigment A, peonidin 3-O-(6″-p-coumaroyl-glucoside), cyanidin 3-O-xylosyl-rutinoside and pelargonidin 3-O-rutinoside. 1-MCP elevated content of anthocyanins, which is benefit for color change in M group. However, NO treatment inhibited most of anthocyanin biosynthesis except peak 1. In addition, NO treatment here inhibits most of the phenolic compounds except peak 1,8,17,18. Intriguingly, the combined treatment showed the similar phenomenon with NO fumigation, but not the 1-MCP treatment. Previous researches such as NO treatment on strawberry [52] and Chinese winter jujube [53] showed that NO fumigation increased total phenolic content, but they did not provide more details about the specific increased or decreased phenolic compounds. In our study, we found that NO application exhibits the strong influence on phenolic biosynthesis, which effect even manifested in MN group. We deduced that NO might act as an internal signal and mediate secondary metabolism in plant cells [54]. However, the underlying mechanism of phenolic compounds and NO needs to be seen.

## 4. Conclusions

The combined 1-MCP and NO treatment showed the best effect on the improvement of postharvest fruit quality by maintaining good physical characteristics, decelerating fruit firmness, inhibiting ROS production, activating antioxidant enzymes and thus, postponing fruit ripening and senescence. NO application can extent peach shelf-life mainly by stimulating antioxidant enzymes. Moreover, NO application showed a greater effect on phenolic synthesis than 1-MCP. Regardless of the mode of action of NO and 1-MCP, for ‘Xiahui-8′ peach, 1-MCP represents a more effective commercial option to inhibit senescence than NO treatment. Treatments with 1-MCP can enhance PAL and POD metabolism by activating via transcription upregulation the expression of *PpaPAL* and *PpaPOD* separately, while playing a lesser role in modulating the expression of *PpaCAT*, *PpaSOD* and *PpaAPX*. MN treatment manifested highest firmness, antioxidant enzymatic activities and lowest ROS content compared with 1-MCP or NO treatment alone. This study provides informative physiological, biochemical and molecular evidence for the benefits of using the combined 1-MCP and NO treatment on peach fruit due to a functional synergy between these two chemical agents.

## Figures and Tables

**Table 1:** Pearson Correlation analysis of enzymatic activity and related gene expression.

	*PpaSOD*/SOD	*PpaCAT*/CAT	*PpaPOD*/POD	*PpaPOD*-1/POD	*PpaAPX*/APX	*PpaPAL*/PAL
Pearson correlation	−0.053	−0.533 **	0.112	−0.406	0.016	0.588 **

** *p* < 0.01.

**Table 2:** Phenolic compounds analyzed by LC/MS.

Peak	Proposed Compounds	Catagory	RT (min)	Neutral Mass (Da)	^a^ [M+H]/^b^ [M-H] (m/z)	Mass Error (Ppm)	Formula	Fragment Number	Relative Amount of Phenolic Compounds ^c^
1	Pigment A	Anthocyanins	4.09	609.1615	610.1688 ^a^	0.1	C31H29O13	22	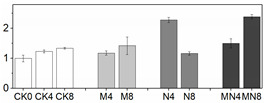
2	Peonidin 3-O-(6″-p-coumaroyl-glucoside)	Anthocyanins	4.82	609.1622	610.1695 ^a^	−0.2	C31H29O13	37	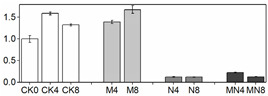
3	Cyanidin 3-O-xylosyl-rutinoside	Anthocyanins	9.64	727.2090	728.2163 ^a^	0.5	C32H39O19	7	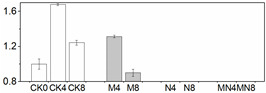
4	Pelargonidin 3-O-rutinoside	Anthocyanins	9.70	579.1731	580.1804 ^a^	1.2	C27H31O14	6	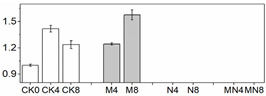
5	Naringenin 4′-O-glucuronide	Flavanones	3.94	448.1003	449.1076 ^a^	0	C21H20O11	27	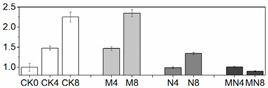
6	Poncirin	Flavanones	9.57	594.1942	595.2014 ^a^	−0.3	C28H34O14	19	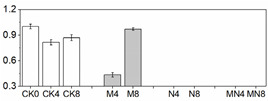
7	(+)-Gallocatechin	Flavanols	4.23	306.0725	307.0797 ^a^	0.1	C15H14O7	6	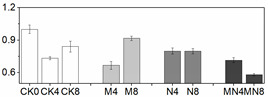
8	(+)-Catechin	Flavanols	3.46	290.0788	291.0861 ^a^	−0.2	C15H14O6	3	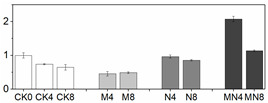
9	Luteolin 7-O-glucuronide	Flavones	0.92	462.0811	463.0883 ^a^	1.4	C21H18O12	17	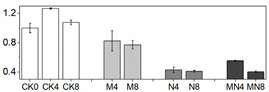
10	Kaempferol	Flavones	3.94	286.0479	287.0738 ^a^	0	C15H10O6	5	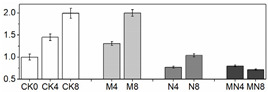
11	Kaempferol 3-O-galactoside	Flavones	3.95	448.1006	449.1079 ^a^	0	C21H20O11	23	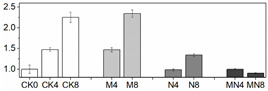
12	Apigenin 7-O-glucoside	Flavones	4.39	432.1066	433.1139 ^a^	0.1	C21H20O10	17	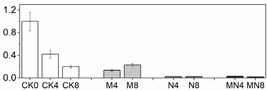
13	Luteolin 7-O-(2-apiosyl-6-malonyl)-glucoside	Flavones	9.48	666.1414	667.1486 ^a^	−2.8	C29H30O18	22	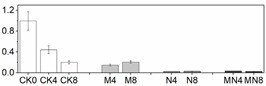
14	5,3′,4′-Trihydroxy-3-methoxy-6:7-methylenedioxyflavone 4′-O-glucuronide	Flavonols	1.32	520.0844	521.0917 ^a^	0.2	C23H20O14	9	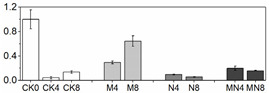
15	Isorhamnetin 3-O-glucoside 7-O-rhamnoside	Flavonols	4.84	624.1701	625.1774 ^a^	1.2	C28H32O16	40	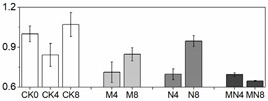
16	Gallic acid	Phenolic acids	2.50	170.0210	169.0156 ^b^	0.1	C7H6O5	2	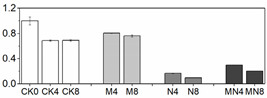
17	4-Hydroxybenzoic acid 4-O-glucoside	Phenolic acids	3.38	323.0734	299.0841 ^b^	−0.2	C13H16O8	10	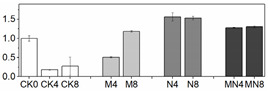
18	3-Caffeoylquinic acid	Phenolic acids	3.55	354.0945	353.1012 ^b^	−0.6	C16H18O9	8	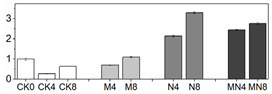
19	3-Feruloylquinic acid	Phenolic acids	4.36	368.1102	367.1101 ^b^	−0.4	C17H20O9	13	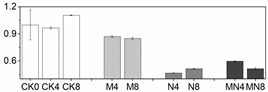
20	Caffeoyl glucose	Phenolic acids	7.44	342.0935	341.0570 ^b^	1	C15H18O9	7	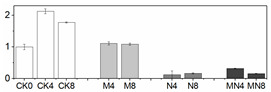

^a^: Positive ionization mode in LC/MS analysis; ^b^: negative ionization mode in LC/MS analysis; ^c^: Relative content of phenolics are expressed according to the peak value of each compound at D0, which are set to 1. Values are the mean ± SE from 3 replicates. CK0, CK4 and CK8 mean samples taken from day 0, day 4 and day 8 of CK group; M4 and M8 mean samples taken from day 4 and day 8 of M group; N4 and N8 mean samples taken from day 4 and day 8 of N group; MN4 and MN8 mean samples taken from day 4 and day 8 of MN group.

## Data Availability

Not applicable.

## Supplementary Materials

The following are available online at https://www.mdpi.com/article/10.3390/foods10122956/s1, Table S1: Primers used for quantification of mRNA levels by qRT-PCR.
