# Supplementary material for: Synergy of Nitric Oxide and 1-Methylcyclopropene Treatment in Prolong Ripening and Senescence of Peach Fruit
Source: Foods. 2021 Dec 1;10(12):2956. doi: 10.3390/foods10122956 (PMC8701743; doi:10.3390/foods10122956)
Supplement: Supplementary file 1 [file foods-10-02956-s001.zip › foods-1458859-supplementary.pdf]

Table S1: Primers used for quantification of mRNA levels by qRT-PCR

Gene	Gene accession	Forward*	Reverse*
SOD	Ppa010748m	CTCACTCAAGTCCAGCAACGCA	GAGAAGGTTTGAAAGCCACGGG
PAL	Ppa002328m	TTGGCTCTGGTTTGGCTTCTAT	TCTGTCTTGCTTAGGCTTCTGA
POD	Ppa010426m	GAAGTTTCCGATTCTCTCATAC	TGGTGGCGGTTCTTGTTTGT
POD-1	Ppa010431m	CTCGTATTTCACGGTGCTCCTG	CGGAAGACAGGGTCAGACAGAA
CAT	Ppa004763m	GGCTTATCTCTGCTCTCTCACC	ACGCTCTGGGATTCGTTCTCTA
APX	Ppa010673m	TTGTGCTCTACTCGTGCCACTCCA	CTTGTACTCTTCGCTCACGGTAGG
TEF2	Ppa001367m	GGTGTGACGATGAAGAGTGATG	TGAAGGAGAGGGAAGGTGAAAG

*Primers are indicated in the 5'→3' direction
